# Developmental dynamics of cellular specialization during proanthocyanidin accumulation in persimmon fruit

**DOI:** 10.1093/plphys/kiaf645

**Published:** 2026-01-30

**Authors:** Yosuke Fujiwara, Soichiro Nishiyama, Akane Kusumi, Keiko Okamoto-Furuta, Hisayo Yamane, Keizo Yonemori, Ryutaro Tao

**Affiliations:** Graduate School of Agriculture, Kyoto University, Kitashirakawa Oiwake-cho, Sakyo-ku, Kyoto, Kyoto 606-8502, Japan; Graduate School of Agriculture, Kyoto University, Kitashirakawa Oiwake-cho, Sakyo-ku, Kyoto, Kyoto 606-8502, Japan; Research Center for Agricultural Information Technology, National Agriculture and Food Research Organization, 1-31-1 Kannondai, Tsukuba, Ibaraki 305-8517, Japan; Graduate School of Agriculture, Kyoto University, Kitashirakawa Oiwake-cho, Sakyo-ku, Kyoto, Kyoto 606-8502, Japan; Graduate School of Medicine, Kyoto University, Yoshida Konoe-cho, Sakyo-ku, Kyoto, Kyoto 606-8501, Japan; Graduate School of Agriculture, Kyoto University, Kitashirakawa Oiwake-cho, Sakyo-ku, Kyoto, Kyoto 606-8502, Japan; Ryukoku Extension Center (REC), Ryukoku University, Seta Oe-cho, Otsu, Shiga 520-2194, Japan; Graduate School of Agriculture, Kyoto University, Kitashirakawa Oiwake-cho, Sakyo-ku, Kyoto, Kyoto 606-8502, Japan

## Abstract

Spatial gene expression and 2D/3D ultrastructural analyses reveal a unique intercellular sequestration mechanism underlying massive proanthocyanidin accumulation in persimmon fruit.

Dear Editor,

Persimmon (*Diospyros kaki*) fruit accumulates exceptionally large amounts of proanthocyanidins (PAs). Because PAs are highly reactive and potentially toxic to the cell, they are often sequestered into compartmentalized structures (Dixon and Sarnala 2020; Lu et al. 2022). In persimmon, PAs are stored in vacuoles of specialized “tannin cells” (TCs), yet the developmental origin and regulation of these cells have remained unclear. Here, we propose an intercellular partitioning mechanism of PA synthesis and sequestration as a basis for massive PA accumulation.

PAs, common secondary metabolites in land plants, are flavan-3-ol polymers that accumulate in vacuoles and confer astringency while providing antioxidant benefits (Dixon et al. 2005; Aron and Kennedy 2008). Although their biosynthesis pathway is well characterized, intracellular trafficking to the vacuole and polymerization remain incompletely resolved (Zhao 2015; Dixon and Sarnala 2020). Oriental persimmon shows extreme PA accumulation, with sequestration in idioblast TCs dispersed in the parenchyma and often clustered. TCs were documented more than a century ago, and their size is known to correlate with fruit PA content (Howard 1906; Tokugawa and Yuasa 1936; Yonemori and Matsushima 1985, 1987; Ikegami et al. 2004; Hamada et al. 2009; Fig. 1A and B). A distinctive feature is the presence of large open pores (OPs) in cell walls (Yonemori and Matsushima 1987). Astringency strongly affects persimmon's market value; pollination-constant and nonastringent (PCNA) mutants (ie ‘Fuyu’ and ‘Hanagosho’) cease PA accumulation early to produce nonastringent fruit (Fig. 1A) and are widely used in cultivation and modern breeding. PCNA is conferred by a recessive allele at the *ASTRINGENCY* (*AST*) locus (Ikeda et al. 1985; Yamada and Sato 2002; Nishiyama et al. 2018), but the causal gene remains unknown.

**Figure 1.**
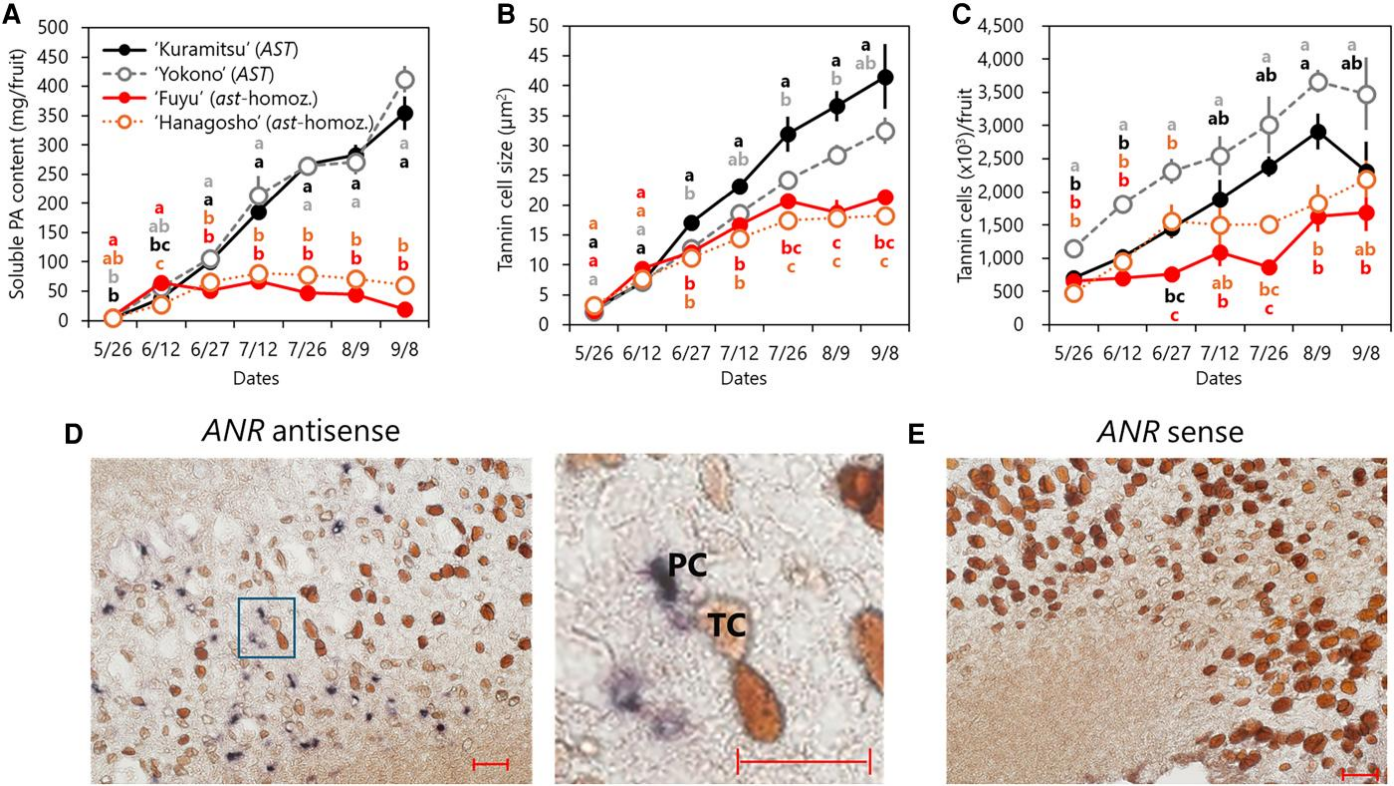
TC development during persimmon fruit development of each cultivar. A) Soluble PA content per fruit in different cultivars (*N* = 5). |B) TC size (*N* = 10). C) Number of TCs per fruit (*N* = 5). Error bars indicate SE. ‘Kuramitsu’ and ‘Yokono’: non-PCNA; ‘Fuyu’ and ‘Hanagosho’: PCNA. Different letters indicate significant differences (*P* < 0.05, Tukey’s honestly significant difference). For a to c), the *x* axis represents the sampling date. D) In situ hybridization for *ANR* in the mesocarp tissue (‘Saijo’; 2 weeks after bloom [WAB]; antisense probe; enlargement of the boxed region shown at the right). E) Sense-probe negative control for the same fruit tissue as shown in panel D). Scale bars in D and E) represent 50 μm. PC, parenchyma cell; TC, tannin cell. In this observation, TCs appeared brown through natural oxidation without any staining reagent.

Here, using an enzymatic and hydraulic workflow (Supplementary Figure S1), we quantified TCs per fruit and observed a steady increase toward the onset of ripening in all cultivars (Fig. 1C). We also observed a positive association between the TC number and the soluble PA content in non-PCNA (‘Kuramitsu’ and ‘Yokono’) and in the relatively PA-rich PCNA cultivar ‘Hanagosho’ (Supplementary Figure S2). These patterns suggest that storage capacity expands via continued TC differentiation during the PA-accumulation window.

In situ hybridization revealed that *anthocyanidin reductase* (*ANR*), a key enzyme that supplies the PA extension unit epigallocatechin in persimmon fruit (Akagi et al. 2009), was expressed in a scattered pattern across the mesocarp and was frequently strongest in parenchyma cells (PCs) directly adjacent to TCs (Fig. 1d and e; Supplementary Figure S3). This pattern indicates functional partitioning: PCs synthesize PA components, while TCs specialize in sequestration. This contrasts with systems where biosynthesis and storage colocalize, such as the *Arabidopsis* seed coat, in which PAs accumulate in the inner integument and *BANYULS* (*ANR* homolog) is expressed in those layers (Debeaujon et al. 2003). Similar colocalization can be seen in different organs across species (Tsai et al. 2006; Abeynayake et al. 2011; Westley et al. 2024). In *Kalanchoe* and *Acorus*, chalcone synthase is restricted to PA-containing idioblasts (Karwatzki et al. 1993), unlike the adjacent parenchyma expression seen here. These examples highlight a distinctive sequestration system in persimmon.

Microscopic observation of OPs in persimmon is challenging: 2D imaging is sensitive to sectioning geometry (ie sectioning must be performed precisely at their location), and ultrathin sectioning is extremely difficult because TCs and PCs differ in physical properties after fixation. We therefore used focused ion beam–scanning electron microscopy (FIB-SEM) to reconstruct interfaces in 3D (Supplementary Videos S1 and S2), complemented by transmission electron microscopy (TEM) to resolve ultrastructure. Methods are provided in the Supplementary Material (Supplementary Table S1).

We observed plasmodesmata (PD) clustered at interfaces between developing TCs and PCs, with the TC vacuole attached to the wall (Fig. 2A and B). Organelles, such as the Golgi apparatus, endoplasmic reticulum (ER), mitochondria, and plastids, are enriched at these interfaces (Supplementary Figure S4). ER abundance varied among TCs (Supplementary Figure S5), consistent with the absence of *ANR* in mature TCs (Fig. 1D) and suggesting that ER-poor cells are storage-oriented. At later stages, OPs interrupting the wall were frequently observed, and vacuoles of adjacent cells were often continuous through OPs (Fig. 2C to F). Vesicle-like structures were frequently associated with PD at TC–PC interfaces (Fig. 2G and H). FIB-SEM enabled OP quantification and showed that OPs were most frequent at TC–TC interfaces and least at PC–PC interfaces, whereas total PD + OP counts were comparable among interface types (Fig. 2I). Nearest-neighbor distances further supported OP enrichment at TC cell walls, since OP spacing was significantly larger in PC–PC interfaces than in interfaces that included TCs in most cultivars tested (Fig. 2J). All these features were observed in both non-PCNA and PCNA (Supplementary Figure S6).

**Figure 2.**
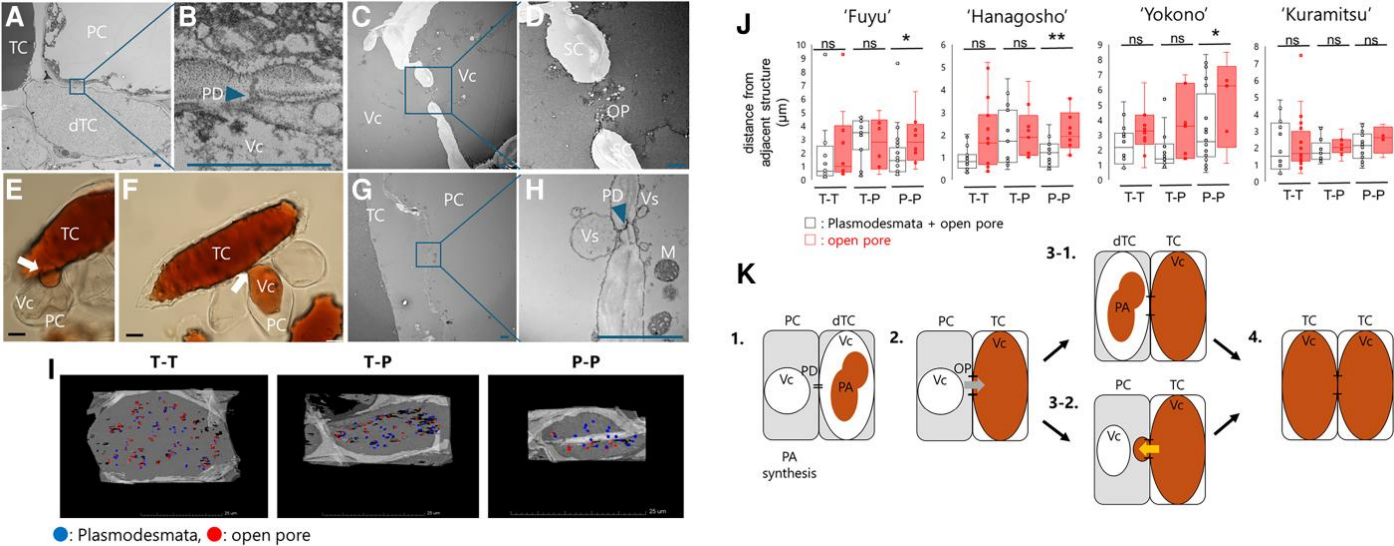
Intercellular interface architecture of TCs. (A) TEM overview of the interface between a developing TC and a PC (‘Fuyu’; 0 WAB). B) Magnified view of A) showing a PD cluster. C) Two adjacent TCs exhibiting an intercellular connection (‘Kuramitsu’; 7 WAB). D) Magnified OP bridging PA-accumulating vacuoles. E and F) Protrusions from mature TCs penetrating adjacent PCs. Isolated cells were observed by light microscopy (‘Kuramitsu’; 17 WAB). G and H) Vesicle-like structures associated with PD at tannin–parenchyma interfaces (‘Kuramitsu’; 7 WAB). In E and F), bars represent 50 µm, and in A to D) and G and H), the blue bar represents 2 µm. I) Visualization of distribution of PD (blue) and OPs (red) across different cell interfaces via FIB-SEM (‘Fuyu’; 0 WAB). ‘T’ and ‘P’ represent TCs and PCs, respectively. Scales represent 25 μm. J) Nearest-neighbor distances for OPs (and PD + OP combined) across 4 cultivars. The boxes represent the interquartile range, the horizontal line inside each box indicates the median, the whiskers show the minimum and maximum values excluding outliers, and the dots represent the values. * and ** denote *P* < 0.05 and *P* < 0.01, respectively, and ns represents nonsignificant difference (1-tailed Student's *t*-tests). K) Schematic model: PD-originated OPs enable transport of PA-related compounds; this promotes differentiation of the neighboring parenchyma into TCs via 2 routes (Stages 3-1/4 and 3-2/4). The arrow from PC to TC represents the transport of PA-related compounds from a PC into the adjacent TC through OPs that originate from PD (indicated by short horizontal lines). The arrow from TC to PC represents the protrusion of PA-containing vacuoles from a TC into the adjacent PC. PC, parenchyma cell; TC/dTC, (developing) tannin cell; Vc, vacuole; PD, plasmodesmata; OP, open pore; SC, secondary cell wall; Vs, vesicle-like structure; M, mitochondria.

We propose two complementary routes for TC expansion active during PA accumulation (Fig. 2K). In one route, PCs adjacent to TCs differentiate into new TCs, consistent with the *ANR* spatial pattern. In the other, TC protrusions penetrate neighboring cells and fuse vacuoles through OPs, yielding clustered TCs with vacuolar continuity. Meanwhile, some *ANR* signals are isolated from existing TCs (Fig. 1D), indicating a de novo path that does not require direct contact. The presence of OPs and interface features in PCNA fruit (Fig. 2J; Supplementary Figure S6) indicates that the anatomical “containers” for massive PA storage can form even when biosynthesis is curtailed, placing the *AST* genes more plausibly on biosynthesis or intracellular transport rather than intercellular sequestration.

Several mechanistic questions follow. First, if biosynthesis is localized, TCs may emit positional cues that induce biosynthesis in adjacent cells; this remains untested. Second, the origin of OPs is unclear; some observations suggest physical rupture, whereas the frequent association of vesicle-like structures and ER at PD points to regulated wall remodeling. Third, isolated *ANR* expression implies an additional initiation path. Addressing these points will clarify how the storage cells are specified and expanded.

We propose that specialized intercellular sequestration enables persimmon to accumulate PAs massively while minimizing cytosolic exposure. This perspective reframes metabolite control as a problem of interface biology as well as pathway flux. The framework suggests targets for breeding or engineering. We anticipate similar strategies may apply to other secondary metabolites that require compartmentalization for plant fitness and product quality.

## Supplementary Material

kiaf645_Supplementary_Data

## Data Availability

The data supporting the findings of this study are available within the article and its Supplementary Materials. Raw FIB-SEM datasets are available from the corresponding author upon reasonable request. The code and trained segmentation models used in this study are publicly available at https://github.com/pomology-ku/tc-fibsem-app.

## Supplementary data

The following materials are available in the online version of this article.

**Supplementary Figure S1**. Schematic workflow for quantifying the number of TCs in persimmon fruit flesh.

**Supplementary Figure S2**. Correlation between the soluble PA content and the number of TCs per fruit in 4 persimmon cultivars.

**Supplementary Figure S3**. In situ hybridization for *ANR* in the flesh around the vertical groove on the fruit surface of ‘Saijo’ at 2 WAB.

**Supplementary Figure S4**. Transmission electron micrographs showing organelles localized at the cellular interface around TCs.

**Supplementary Figure S5**. Organelle observation in developing TCs.

**Supplementary Figure S6**. The characteristics of the cellular interface around TCs in PCNA mutants.

**Supplementary Table S1**. Data acquisition parameters for FIB-SEM.

**Supplementary Video S1**. Sequential FIB-SEM images of the fruit flesh tissue of ‘Fuyu’ at flowering.

**Supplementary Video S2**. 3D rendering of segmented FIB-SEM images of the fruit flesh tissue of ‘Fuyu’ at flowering.

## Dive Curated Terms

The following phenotypic, genotypic, and functional terms are of significance to the work described in this paper:
