# Supplementary material for: Developmental dynamics of cellular specialization during proanthocyanidin accumulation in persimmon fruit
Source: Plant Physiol. 2026 Jan 30;200(1):kiaf645. doi: 10.1093/plphys/kiaf645 (PMC12857211; doi:10.1093/plphys/kiaf645)
Supplement: kiaf645_Supplementary_Data [file kiaf645_supplementary_data.zip › Supplementary data v5.pdf]

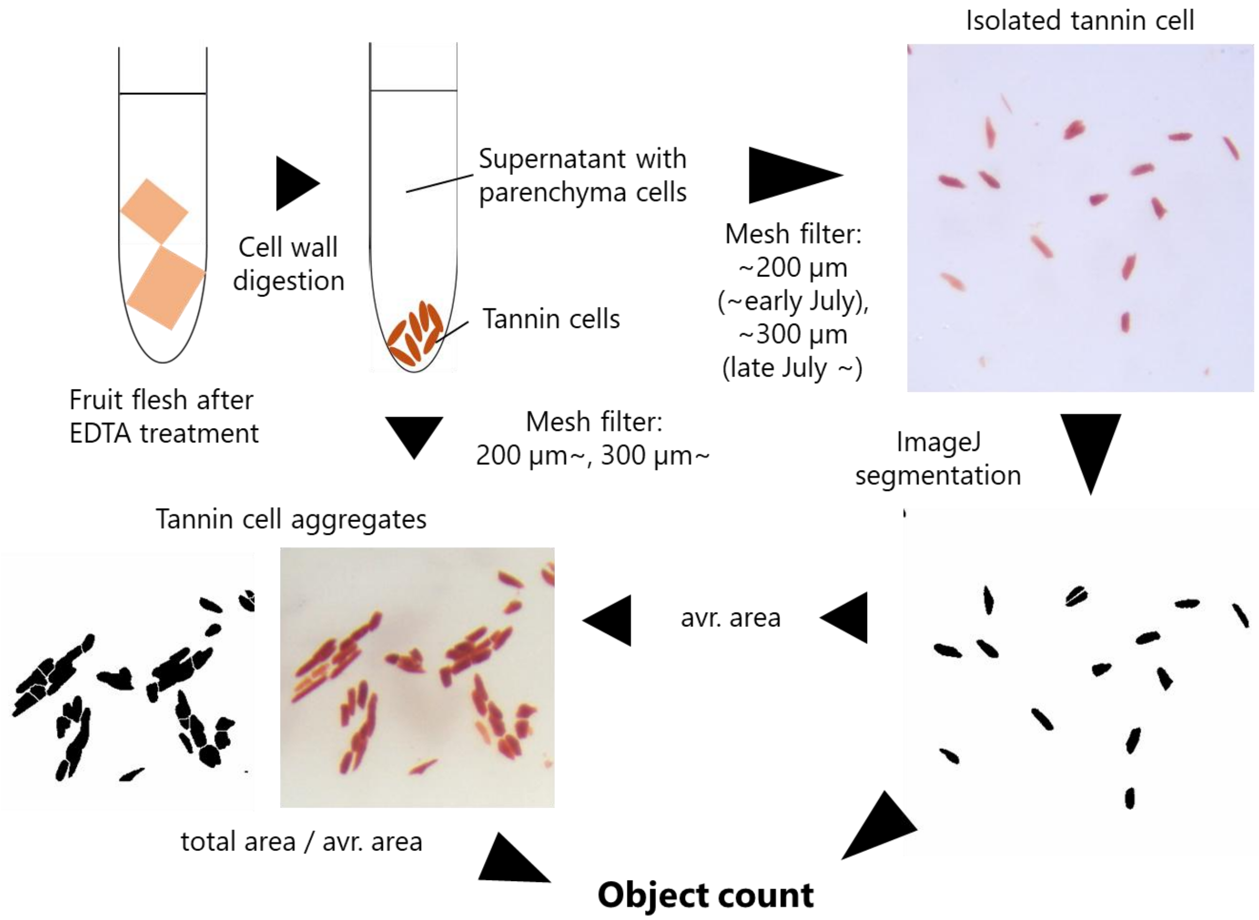

Supplementary Figure S1. Schematic workflow for quantifying the number of tannin cells in persimmon fruit flesh. Some tannin cells form tightly bound aggregates, making it difficult to isolate and count individual cells accurately. In this workflow, the tannin cell slurry was passed through a mesh filter to separate isolated tannin cells from aggregates. Both isolated cells and aggregates were then analyzed separately using image analysis. For aggregates, the number of tannin cells was estimated by dividing the total area of each aggregate by the average area of an individual cell.

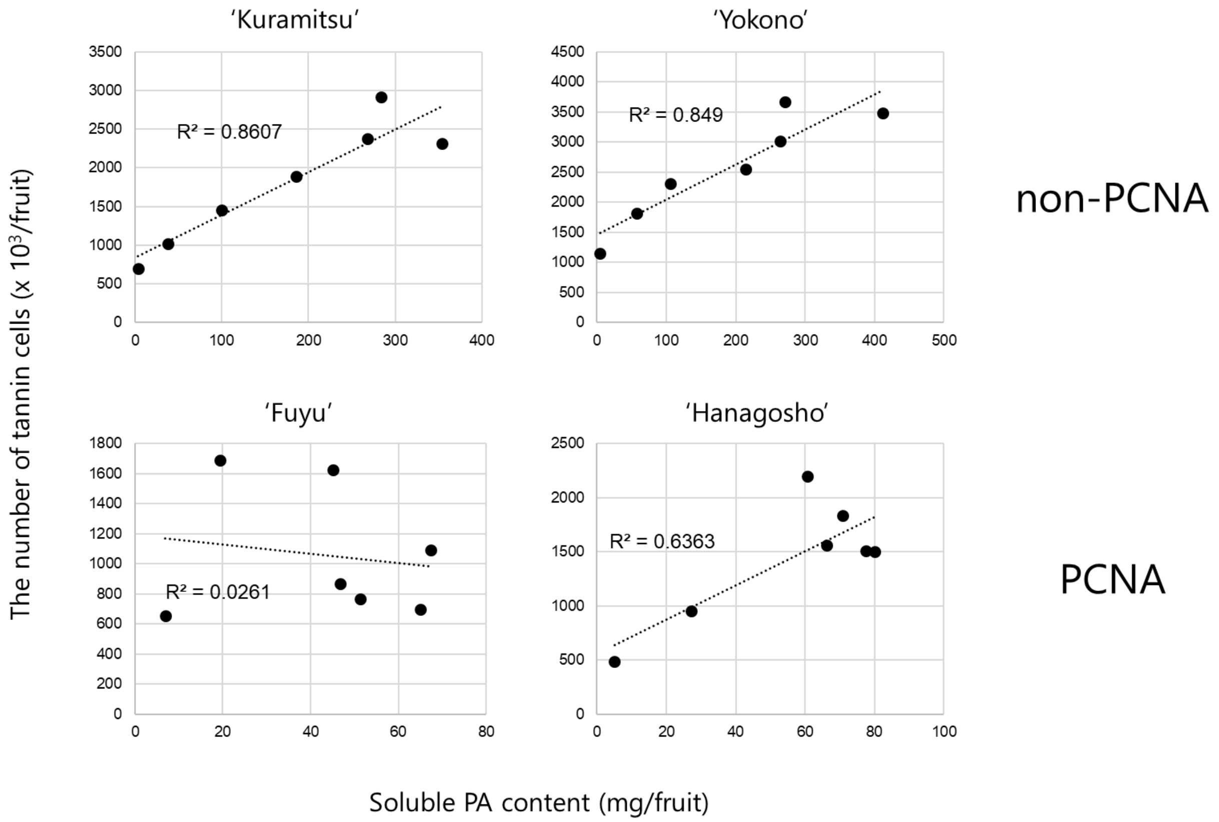

Supplementary Figure S2. Correlation between soluble PA content and the number of tannin cells per fruit in four persimmon cultivars. Data points were retrieved from seasonal measurements at seven developmental stages, as analyzed in Figure 1.

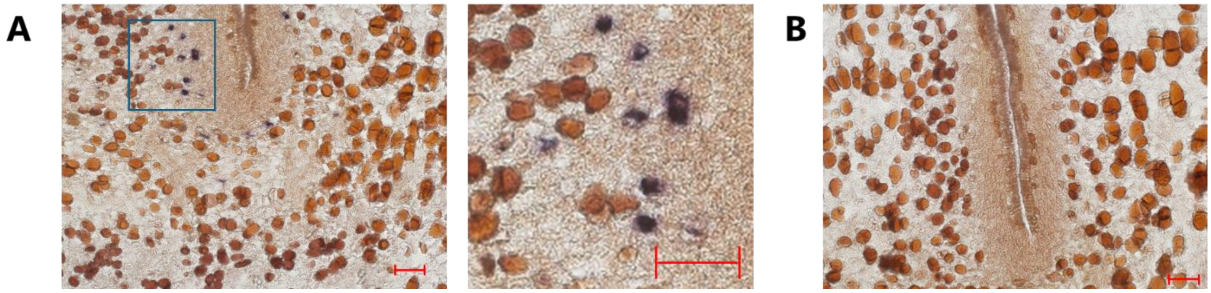

Supplementary Figure S3. *In situ* hybridization for *anthocyanidin reductase* (*ANR*) in the flesh around the vertical groove on the fruit surface of 'Saijo' at 2 weeks after bloom (WAB). (A) Dectected signals using an antisense probe. Boxed region shown at right. (B) Sense-probe negative control for the same tissue. Red scale bar, 50 µm.

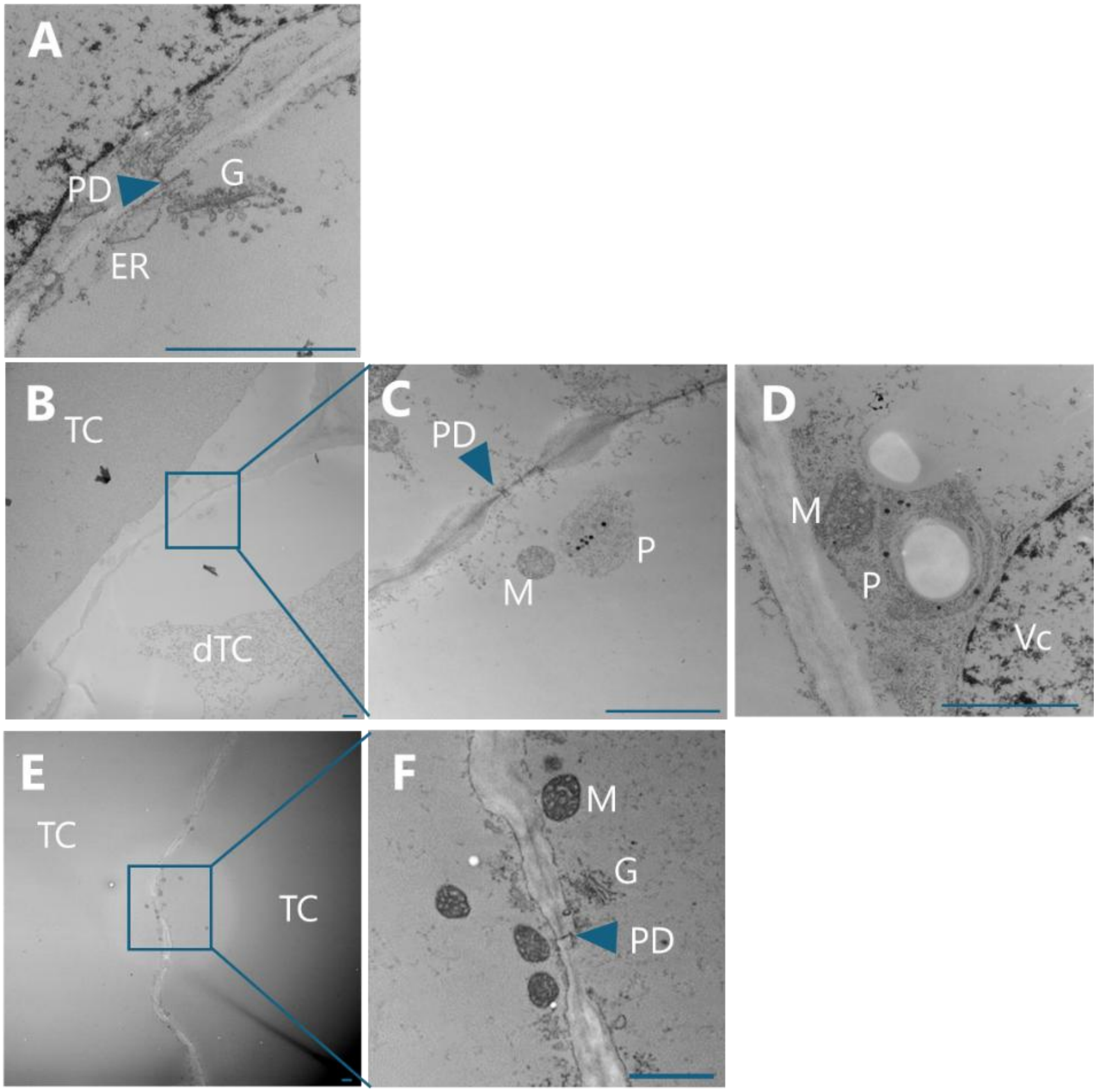

Supplementary Figure S4. Transmission electron micrographs showing organelles localized at the cellular interface around tannin cells. Samples in panels (A) were prepared from 'Fuyu' (PCNA) fruit collected on 1 WAB; panels (B-F) from 'Kuramitsu' (non-PCNA) fruit collected on 7 WAB. Panels (C) and (F) represent the magnified view of (B) and (E), respectively. TC, tannin cell; dTC, developing tannin cell; PD, plasmodesmata; ER, endoplasmic reticulum; G, Golgi apparatus; P, plastid; M, mitochondria; Vc, vacuole. Blue bars represent 2 µm.

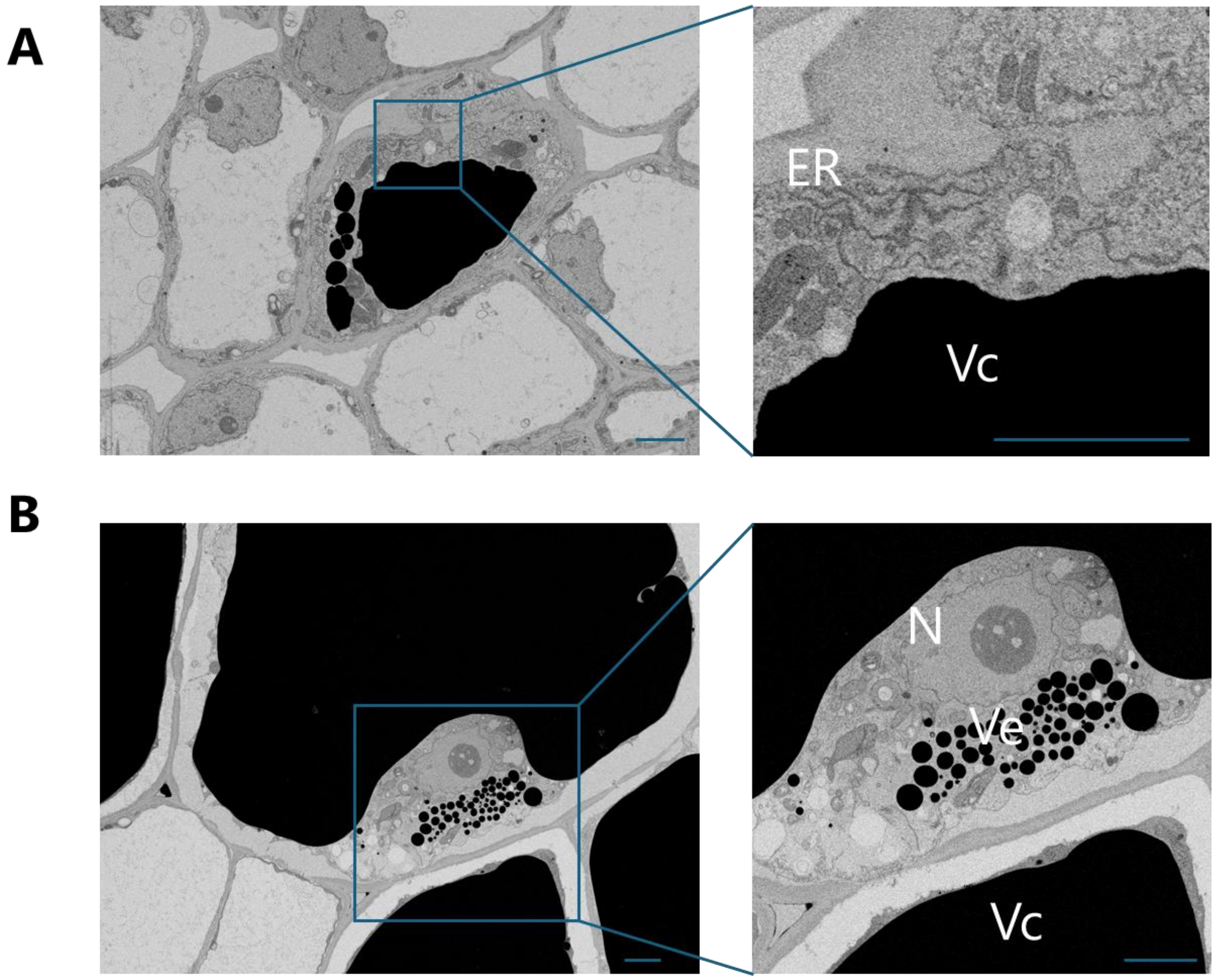

Supplementary Figure S5. Organelle observation in developing tannin cells. Images were obtained from view of serial section observed by FIB-SEM. (A) A tannin cell possessing well-developed ER. Sample of ‘Kuramitsu’ fruit sampled at anthesis was analyzed. (B) A tannin cell in later developing stage without extensive ER development. In contrast, numerous PA-containing small vesicles were observed around the nucleus. In the lower part, a mature tannin cell that had completed PA accumulation and lacked both vesicles and well-developed ER was also observed. Sample of ‘Yokono’ fruit collected at 1 WAB was analyzed. ER, endoplasmic reticulum; Vc, vacuole; N, nucleus; Ve, vesicle-like structure. Bars represent 5 µm.

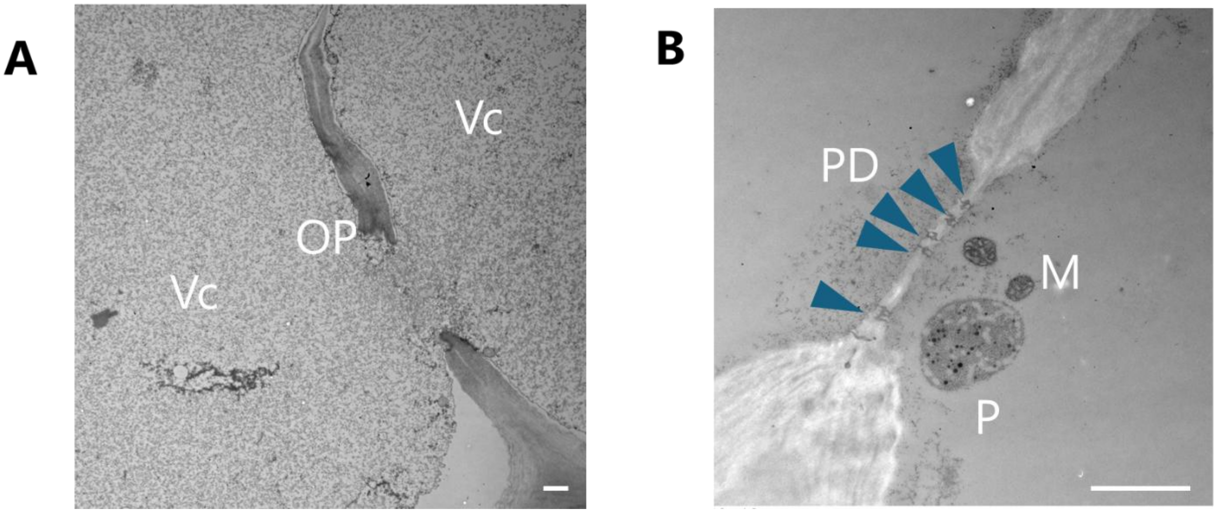

Supplementary Figure S6. The characteristics of cellular interface around tannin cell in PCNA mutants. Samples were prepared from 'Hanagosho' fruit collected at 7 WAB. (A) Large OP and (B) organelles accumulating around PD site were observed by TEM micrograph. Vc, Vacuole; OP, open pore; PD, plasmodesmata; P, plastid; M, mitochondria. Bars represent 2 μm.

Supplementary Table S1. Data acquisition parameters for FIB-SEM

Cultivar	stage	image pixel size (nm)	milling step (nm)
Fuyu	0 WAB	40	100
Hanagosho	1 WAB	30	150
Kuramistu	0 WAB	30	150
Yokono	1 WAB	40	100
