# Supplementary material for: Developmental dynamics of cellular specialization during proanthocyanidin accumulation in persimmon fruit
Source: Plant Physiol. 2026 Jan 30;200(1):kiaf645. doi: 10.1093/plphys/kiaf645 (PMC12857211; doi:10.1093/plphys/kiaf645)
Supplement: kiaf645_Supplementary_Data [file kiaf645_supplementary_data.zip › Supplementary Text.pdf]

Developmental dynamics of cellular specialization during proanthocyanidin accumulation in persimmon fruit

Yosuke Fujiwara[1,*], Soichiro Nishiyama[1,2,*,**], Akane Kusumi[1], Keiko Okamoto-Furuta[3], Hisayo Yamane[1], Keizo Yonemori[4] and Ryutaro Tao[1]

[1] Graduate School of Agriculture, Kyoto University, Kitashirakawa Oiwake-cho, Sakyo-ku, Kyoto 606-8502, Japan
[2] Research Center for Agricultural Information Technology, National Agriculture and Food Research Organization, Tsukuba, Ibaraki 305-8517, Japan
[3] Graduate School of Medicine, Kyoto University, Yoshida Konoe-cho, Sakyo-ku, Kyoto 606-8501, Japan
[4] Ryukoku Extension Center (REC), Ryukoku University, Seta Oe-cho, Otsu, Shiga 520-2194, Japan

*These authors contributed equally to this work.
**Correspondence: nishiyama.soichiro.8e@kyoto-u.ac.jp; +81-75-753-6051

Supplementary Methods

Plant materials

All plant materials were cultivated at the Kyoto Farmstead of the Experimental Farm, Kyoto University (Japan), under conventional cultivation practices. Sampling methods are described in the respective experimental sections below.

PA and tannin cell measurements

Persimmon fruits were collected at seven developmental stages from May to September from two non-PCNA cultivars (‘Kuramitsu’ and ‘Yokono’) and two PCNA cultivars (‘Fuyu’ and ‘Hanagosho’). Five fruits were harvested from each cultivar at each stage, with each fruit treated as a biological replicate. After recording fruit weight, only the mesocarp tissue was excised. For tannin cell analysis, approximately 300 mg of mesocarp tissue was fixed in FAA solution (acetic acid: formalin: water: ethanol = 1:2:7:10) and stored at 4°C. For soluble PA analysis, mesocarp tissue was frozen in liquid nitrogen and stored at –80°C. The quantification of soluble PA was performed according to the method described by Fujiwara et al. (2025).

The fixed mesocarp tissue was immersed in 0.05 M EDTA solution (pH 10.0) and incubated overnight at 50°C. The softened tissue was then transferred to an enzymatic solution (pH 5.0) containing the cell wall degrading enzymes ONOZUKA RS (Yakult, Tokyo, Japan) (0.5%) and Macerozyme R-10 (Yakult) (0.05%). After gently dissociating the tissue with a fine brush, it was incubated at 45°C for 3 hours. The supernatant containing dissociated parenchyma cells was discarded, and the remaining suspension was used for the measurement of tannin cell number and size.

Due to the presence of tightly bound tannin cell aggregates that could not be dissociated by enzymatic treatment, isolated cells and aggregates were quantified separately. Tissue was passed through a stainless-steel mesh chosen for each developmental stage: a 200 µm mesh for samples collected up to 12 July and a 300 µm mesh for samples collected from 21 July onward.

To isolate tannin cell aggregates, distilled water was passed through the mesh to apply hydraulic pressure. Individually dissociated tannin cells and aggregated clusters were collected and stored as 5 mL and 1 mL cell suspensions, respectively. For size analysis, a 50 μL aliquot of the tannin cell suspension was placed onto a glass slide, covered with a coverslip, and observed under a light microscope. Images were recorded for each sample and analyzed using ImageJ (https://imagej.net/ij/index.html) to calculate the area of individual tannin cells. The mean area was determined from measurements of ten cells per sample. For count analysis, first, the number of isolated cells in each image was counted, and the average pixel area per cell was calculated. The total pixel area of each tannin cell aggregate was then measured in 25 μL aliquot of the suspension, and the number of cells per aggregate was estimated by dividing this value by the single-cell mean. For each sample, the numbers of isolated cells and aggregate-derived cells were summed. Finally, using the measured fruit weight, the number of tannin cells per fruit was calculated. These method for tannin cell measurement is summarized in Supplementary Fig. S1.

In situ hybridization

The full-length CDS of *DkANR* was cloned into the pGEM-T Easy Vector. The T7 promoter sequence (5'-TAATACGACTCACTATAGGG-3') was added to the 5' ends of the primers including start and stop codons, and PCR amplification was performed (F: 5'-TAATACGACTCACTATAGGG+ATGGCAGCAGCCCCACCG-3', R:5'-TAATACGACTCACTATAGGG+AGTTCTTCAAAATCCCCTTGGC-3'). The resulting PCR product, containing the T7 promoter at both ends of the full-length CDS, was purified and used as a template. Following the manufacturer's instructions for the DIG RNA Labeling Kit (Roche, Switzerland), sense and antisense DIG-labeled RNA probes were synthesized using T7 RNA polymerase. The probes were used at approximately 1:10 dilution.

Fruits of the persimmon cultivar 'Saijo' were collected on 22 May. The samples were immersed in FAA fixative (ethanol : water : acetic acid : formaldehyde = 9 : 9 : 1 : 1), degassed on ice for at least 30 minutes, and then incubated at 4 °C for 4 hours. After fixation, the samples were dehydrated through a graded ethanol series at 4 °C, and incubated overnight in 100% ethanol at 4 °C. Subsequently, the samples underwent two additional 20-minute washes in 100% ethanol at room temperature, followed by a graded replacement with t-butanol/ethanol. Paraplast Plus (Sigma-Aldrich, US) was then introduced, and samples were incubated overnight at 64 °C to allow t-butanol evaporation. The samples were stored at 4 °C. To ensure complete infiltration of paraffin and evaporation of residual t-butanol, the samples were subjected to twice-daily paraffin changes at 60 °C for approximately one week before being embedded in paraffin.

Transverse sections of 12 μm-thick were prepared from the paraffin blocks using a microtome. The sections were deparaffinized using Lemosol (FUJIFILM Wako, Japan), followed by rehydration through a graded ethanol series. Proteinase K digestion was performed in 100 mM Tris-HCl and 50 mM EDTA buffer at 37 °C for 30 minutes (the working concentration was 144 mU/mL). Acetylation was then conducted in the solution of 13.32 μL/mL triethanolamine and 5 μL/mL acetic anhydride for 10 minutes. The slides were subsequently dehydrated through an ethanol series and vacuum dried.

Hybridization buffer was prepared for each RNA probe with the following components: 100 mg of dextran sulfate (Sigma-Aldrich, US), 439 μL of DEPC-treated water, 60 μL of 5 M NaCl, 10 μL of 1 M Tris-HCl (pH 8.0), 10 μL of 500 mM $NaPO_4$ (pH 8.0), 2 μL of 500 mM EDTA (pH 8.0), 400 μL of formamide, 25 μL of 10% SDS, 20 μL of 50× Denhardt's solution (FUJIFILM Wako, Japan), 5 μL of 25 mg/mL Yeast tRNA (Thermo Fisher Scientific, US). 12.5 μL of

10 mg/mL salmon sperm DNA (Thermo Fisher Scientific, US), 16 µL of RNA probe. The probe solution was incubated at 80 °C for 10 minutes, and 1 µL of RNaseOUT™ Recombinant Ribonuclease Inhibitor (Thermo Fisher Scientific, US) was added. Hybridization was performed by applying the probe solution onto the sections and incubating at 50 °C overnight.

After hybridization, the sections were washed twice in 4× SSC for 10 minutes each. RNaseA treatment (NIPPON GENE, Japan, 20 µg/mL in NTE buffer: 500 mM NaCl, 10 mM Tris-HCl pH 7.5, 1 mM EDTA) was carried out at 37 °C for 30 minutes. Following this, slides were washed three times for 15 minutes in NTE buffer, twice for 20 minutes in 2× SSC, and twice for 20 minutes in 0.5× SSC. Subsequently, the sections were incubated in 100 mM maleic acid and 150 mM NaCl buffer (pH 7.5) for 5 minutes and blocked with 1.5× blocking reagent solution (Roche, Switzerland) for 30 minutes. Anti-digoxigenin-AP, Fab fragments (Roche, Switzerland), diluted 1:1000 in 1.5× blocking solution, were applied and incubated for 60 minutes. The slides were washed twice for 10 minutes each in the same maleic acid buffer and equilibrated in 100 mM Tris-HCl and 100 mM NaCl buffer (pH 9.5) for 5 minutes. Color development was carried out using BCIP-NBT solution (Ready To Use) (Nacalai Tesque, Japan) at room temperature for approximately 20 hours. The reaction was stopped by washing with distilled water. The samples were then mounted using G-Mount (Genostaff, Japan) and observed under an optical microscope.

TEM and FIB-SEM

Fruit fresh was collected at two developmental stages (0-1 and 7-8 WAB) from two non-PCNA cultivars ('Kuramitsu' and 'Yokono') and two PCNA cultivars ('Fuyu' and 'Hanagosho'), which were the same cultivars used for tannin cell measurement. Tissue blocks of approximately 1 x 1 x 2 cm were fixed in 4% PFA and 2% glutaraldehyde in PBS and stored at 4 °C until observation.

For TEM observation, the fixed tissues were washed three times with 0.1 M PBS, then incubated overnight at 4 °C in 1% osmium tetroxide (OsO_4) in 0.1 M PBS. Following this, the samples were washed three more times with 0.1 M PBS, then dehydrated through a graded ethanol series (20%, 30%, 40%, 50%, 60%, 70%, 80%, 90%, 99%, and twice in 100%), followed by two changes of 100% propylene oxide. The samples were then infiltrated with a graded series of propylene oxide:Epon 812 mixtures (5:1, 3:1, 1:1, 1:3), followed by pure resin, and finally polymerized. Ultrathin sections of 70-nm thickness were cut with a diamond knife and stained with uranyl acetate andlead citrate. Samples were observed by TEM (H-7650, Hitachi, Japan).

For FIB-SEM observation, samples were prepared by a modified Ellisman method (West et al, 2010) as described in Katsuno et al (2019), with some modifications. The fixed tissues were first washed three times in distilled water (DW) for 2 minutes each, and then incubated on ice for 3 hours in a solution of 1.5% potassium ferrocyanide and 2% osmium tetroxide. After incubation, the samples were washed five times in DW (2 minutes each), followed by post-fixation in 2% osmium tetroxide in DW at room temperature for 2 hours. The samples were then washed again five times in DW (2 minutes each) and block-stained with 1% uranyl acetate at 4 °C overnight. After another five DW washes (2 minutes each), lead staining was performed using the aspartic acid–lead method at 60 °C for 30 minutes. The samples were washed again five times in DW and then dehydrated and embedded in Epon 812 using the same procedure as for TEM.

The FIB-SEM observation was done as described in Katsuno et al (2019). The specimens were set on the stage of a FIB-SEM (CrossBeam 540, Zeiss, Germany). Microfabrication around the reconstruction area was performed as previously described (Ohta et al, 2012). A sample-specific number of serial images were acquired through repeated cycles of sample surface milling

and imaging using SmartFIB (ZEISS), resulting in a dataset with a depth of approximately 50 µm. Milling was performed using a gallium ion beam at 30 kV with a current of either 15nA. Other imaging parameters were as follows: scan speed = 45.1 sec/image image resolution = 2048 × 1536 pixels. Milling pitch and pixel size were adjusted for each sample and summarized in Supplementary Table S1.

The acquired images were registered for each sample using the drift correction function in Microscopy Image Browser ver 2.81 (Belevich et al., 2016) with default parameters. OP and PD were manually annotated, and distances between each structure and its nearest neighbor were measured for at least five instances of each interface. Structures with a dark line crossing the cell wall were labeled as PD, while those showing a complete break in the cell wall were labeled as OP. It should be noted that, under our microfabrication and visualization parameters, it is difficult to capture slices directly through the center of PDs, and thus not all PDs are likely to have been detected. Therefore, the measured distances may be longer than the actual physical distances. Segmentation was performed using a custom app built on MONAI Label (Diaz-Pinto et al., 2022). We used a 2.5D segmentation approach, in which each slice was input as a three-channel image by stacking it with the adjacent slices, to generate segmentation labels. For this, we utilized the segmentation_models.pytorch library (Iakubovskii, 2019; https://github.com/qubvel/segmentation_models.pytorch) to implement EfficientNet-B4 (Tan and Le, 2019) as the encoder backbone and fine-tuned the model to segment cell walls. Tannin cell vacuoles were segmented based on signal intensity. All segmentation and annotation results were visualized in 3D Slicer (Kikinis et al., 2014). The code and trained segmentation models have been made publicly available at [https://github.com/pomology-ku/tc-fibsem-app].

Author contributions

SN, KY, and RT conceived the study. SN designed the experiments. YF and SN conducted most of the experiments and analyses, with support from the other authors. AK optimized the *in situ* hybridization imaging protocol and led the visualization. KF optimized the TEM and FIB-SEM protocols, prepared the specimens, and assisted in acquiring the micrographs. SN, HY, and RT prepared the plant materials and experimental facilities. YF and SN wrote the manuscript. All authors read and approved the final version.

References

Fujiwara Y, Nishiyama S, Onoue N, Kono A, Sato A, Yonemori K, Tao R (2025) Phenotypic characterization of astringent persimmon lines with recessive homozygosity at the *ASTRINGENCY* marker locus. Hort J 94: 148–161

West JB, Fu Z, Deerinck TJ, Mackey MR, Obayashi JT, Ellisman MH (2010) Structure-function studies of blood and air capillaries in chicken lung using 3D electron microscopy. Respir Physiol Neurobiol 170: 202–209

Katsuno T, Belyantseva IA, Cartagena-Rivera AX, Ohta K, Crump SM, Petralia RS, Ono K, Tona R, Imtiaz A, Rehman A, et al (2019) TRIOBP-5 sculpts stereocilia rootlets and stiffens supporting cells enabling hearing. JCI Insight. doi: 10.1172/jci.insight.128561

Ohta K, Sadayama S, Togo A, Higashi R, Tanoue R, Nakamura K-I (2012) Beam deceleration for block-face scanning electron microscopy of embedded biological tissue. Micron 43: 612–620

Belevich I, Joensuu M, Kumar D, Vihinen H, Jokitalo E (2016) Microscopy image browser: A platform for segmentation and analysis of multidimensional datasets. PLoS Biol 14: e1002340

Diaz-Pinto A, Alle S, Nath V, Tang Y, Ihsani A, Asad M, Pérez-García F, Mehta P, Li W, Flores M, et al (2024) MONAI Label: A framework for AI-assisted interactive labeling of 3D medical images. Med Image Anal 95: 103207

Iakubovskii, P (2019) Segmentation Models Pytorch. GitHub repository. https://github.com/qubvel/segmentation_models.pytorch

Tan M, Le QV (2019) EfficientNet: Rethinking model scaling for convolutional Neural Networks. ICML abs/1905.11946: 6105–6114

Kikinis R, Pieper SD, Vosburgh KG (2014) 3D slicer: A platform for subject-specific image analysis, visualization, and clinical support. Intraoperative Imaging and Image-Guided Therapy. Springer New York, New York, NY, pp 277–289
