# Supplementary material for: Developmental dynamics of cellular specialization during proanthocyanidin accumulation in persimmon fruit
Source: Plant Physiol. 2026 Jan 30;200(1):kiaf645. doi: 10.1093/plphys/kiaf645 (PMC12857211; doi:10.1093/plphys/kiaf645)
Supplement: kiaf645_Supplementary_Data [file kiaf645_supplementary_data.zip › Supplementary video legends v1.pdf]

Supplementary Video S1. Sequential FIB-SEM images of the fruit flesh tissue of ‘Fuyu’ at flowering. Scale bar represents 5 µm.

Supplementary Video S2. 3D rendering of segmented FIB-SEM images of the fruit flesh tissue of ‘Fuyu’ at flowering. Cell walls are shown in gray and tannin cell vacuoles in red. Scale bar represents 25 µm.
